# Supplementary material for: Optogenetics and electron tomography for structure-function analysis of cochlear ribbon synapses
Source: eLife. 2022 Dec 23;11:e79494. doi: 10.7554/eLife.79494 (PMC9908081; doi:10.7554/eLife.79494)
Supplement: Supplementary file 1. — Statistics could not be performed due to the rare abundance of CC structures at the AZ in our tomograms. The gray columns indicate CC structures intermingled with the membrane-proximal (MP)-synaptic vesicle (SV) pool. [file elife-79494-supp1.docx]

**Supplementary file 1**

| \| **Condition &  Genotype** \| ***N*** \| ***n of ribbons* with CC structures/total n** \| **Clathrin-coated (CC) structures** \| \| \| \| \| \| \| --- \| --- \| --- \| --- \| --- \| --- \| --- \| --- \| --- \| \| **0 – 100 nm** \| **100 – 200 nm** \| \| \| **200 - 500 nm** \| \| \| CCVs \| Invagina-tions \| CCVs \| Invagina-tions \| CCVs \| Invagina-tions \| \|  \|  \|  \| (y=Distance to membrane, x=Distance to PD, d=Diameter in nm) \| \| \| \| \| \| \| ChR2ShortStim \| 1 \| 1/11 ribbons \| - \| - \| - \| - \| y=38  x=455  d=121 \| - \| \|  \|  \| 2/11 ribbons \| - \| - \| - \| - \| y=54  x=498  d=102 \| - \| \| ChR2LongStim \| 4 \| 1/26 ribbons \| - \| - \| - \| x=160 \| y=48  x=345  d=69 \| - \| \|  \|  \| 2/26 ribbons \| - \| - \| y=78  x=144  d=109 \| - \| - \| - \| \|  \|  \| 1/26 ribbons \| - \| - \| - \| - \| - \| x=315 \| \| LongStim  B6J \| 2 \| 1/15 ribbons \| - \| - \| y=8 x=126 d=107 \| - \| - \| - \| \|  \|  \| 2/15 ribbons \| - \| x=80 \| y=30 x=185 d=94 \| - \| - \| - \| \|  \|  \| 3/15 ribbons \| - \| - \| y=8 x=170 d=74 \| - \| - \| - \| \| ChR2 NoLight \| 4 \| 1/17 ribbons \| y=48 x=90 d=63 \| - \| - \| - \| - \| - \| |
| --- | --- | --- | --- | --- | --- | --- | --- | --- | --- | --- | --- | --- | --- | --- | --- | --- | --- | --- | --- | --- | --- | --- | --- | --- | --- | --- | --- | --- | --- | --- | --- | --- | --- | --- | --- | --- | --- | --- | --- | --- | --- | --- | --- | --- | --- | --- | --- | --- | --- | --- | --- | --- | --- | --- | --- | --- | --- | --- | --- | --- | --- | --- | --- | --- | --- | --- | --- | --- | --- | --- | --- | --- | --- | --- | --- | --- | --- | --- | --- | --- | --- | --- | --- | --- | --- | --- | --- | --- | --- | --- | --- | --- | --- | --- | --- | --- | --- | --- | --- | --- | --- | --- | --- | --- | --- | --- | --- | --- | --- | --- | --- |

**List of clathrin-coated (CC) structures at the active zone**

Statistics could not be performed due to the rare abundance of CC structures at the AZ in our tomograms. The grey columns indicate CC structures intermingled with the MP-SV pool.
