# Supplementary material for: Optogenetics and electron tomography for structure-function analysis of cochlear ribbon synapses
Source: eLife. 2022 Dec 23;11:e79494. doi: 10.7554/eLife.79494 (PMC9908081; doi:10.7554/eLife.79494)
Supplement: Supplementary file 2. — Data are presented as mean ± SEM. Data was tested for significant differences by one-way ANOVA followed by Tukey’s test (parametric data) or Kruskal-Wallis (KW) test followed by Dunn’s test (non-parametric data). Significant results are indicated with *p<0.05; **p<0.05; and ****p<0.0001. [file elife-79494-supp2.docx]

**Supplementary file 2**

|  | | **B6J Light** | **ChR2 NoLight** | **ChR2 ShortStim** | **ChR2 LongStim** | **Adjusted p-value** | **Test** |
| --- | --- | --- | --- | --- | --- | --- | --- |
| *N*_animals_ | | 1 | 4 | 1 | 3 |  |  |
| *n*_ribbons_ Total | | 9 | 17 | 11 | 21 |  |  |
| *n*_ribbons_ Ai32VC | |  | 9 | 11 | 10 |  |  |
| *n*_ribbons_ Ai32KI | |  | 8 | 0 | 11 |  |  |
| *n*_SV_ Total | | 238 | 576 | 324 | 700 |  |  |
| RA-SVs count | | 26.44 | 37.35 | 29.45 | 33.33 | n.s | ANOVA - Tukey's test |
|  |  | ± 3.296 | ± 3.683 | ± 4.059 | ± 2.372 |  |  |
| Proximal RA-SVs count | | 9.22 | 13.29 | 10.82 | 12.86 | n.s | KW Test - Dunn's test |
|  |  | ± 1.289 | ± 1.928 | ± 1.71 | ± 0.974 |  |  |
| Distal RA-SVs count | | 17.22 | 24.06 | 18.64 | 20.48 | n.s | ANOVA - Tukey's test |
|  |  | ± 2.284 | ± 2.237 | ± 2.577 | ± 1.573 |  |  |
| Diameter of RA-SVs | Proximal pool RA-SVs | 51.09 | 49.51 | 50.00 | 48.98 | B6J Light vs. ChR2 LongStim | ANOVA - Tukey's test |
|  |  | ± 0.513 | ± 0.491 | ± 0.369 | ± 0.337 | 0.0003 |  |
|  |  |  |  |  |  | B6J Light vs. ChR2 NoLight |  |
|  |  |  |  |  |  | 0.0077 |  |
|  |  |  |  |  |  | ChR2 LongStim vs. ChR2 ShortStim |  |
|  |  |  |  |  |  | 0.0169 |  |
|  | Distal pool RA-SVs | 53.66 | 50.36 | 50.39 | 49.69 | B6J Light vs. ChR2 LongStim | KW Test - Dunn's test |
|  |  | ± 0.93 | ± 0.48 | ± 0.315 | ± 0.363 | <0.0001 |  |
|  |  |  |  |  |  | B6J Light vs. ChR2 NoLight |  |
|  |  |  |  |  |  | <0.0001 |  |
|  |  |  |  |  |  | B6J Light vs. ChR2 ShortStim |  |
|  |  |  |  |  |  | 0.0092 |  |
|  |  |  |  |  |  | ChR2 LongStim vs. ChR2 ShortStim |  |
|  |  |  |  |  |  | 0.0022 |  |
| Fraction of SVs without filaments | | 0.27 | 0.32 | 0.21 | 0.23 | n.s | KW Test - Dunn's test |
|  |  | ± 0.027 | ± 0.034 | ± 0.017 | ± 0.035 |  |  |
| Fraction of Ribbon attached SVs | | 0.28 | 0.22 | 0.13 | 0.13 | B6J Light vs. ChR2 LongStim | KW Test - Dunn's test |
|  |  | ± 0.053 | ± 0.026 | ± 0.028 | ± 0.019 | 0.0173 |  |
| Fraction of interconnected SVs | | 0.31 | 0.29 | 0.45 | 0.27 | ChR2 LongStim vs. ChR2 ShortStim | KW Test - Dunn's test |
|  |  | ± 0.051 | ± 0.023 | ± 0.045 | ± 0.038 | 0.0334 |  |
|  |  |  |  |  |  | ChR2 NoLight vs. ChR2 ShortStim |  |
|  |  |  |  |  |  | 0.0266 |  |
| Fraction of Ribbon-attached and interconnected SVs | | 0.14 | 0.17 | 0.20 | 0.14 | n.s | KW Test - Dunn's test |
|  |  | ± 0.029 | ± 0.026 | ± 0.023 | ± 0.026 |  |  |
|  |  |  |  |  |  |  |  |

**List of RA-SV parameters showing the mean ± SEM values, N, n, *p*-values and the statistical tests applied**

Data are presented as mean ± SEM. Data was tested for significant differences by one-way ANOVA followed by Tukey’s test (parametric data) or KW test followed by Dunn’s test (non-parametric data). Significant results are indicated with * p< 0.05; ** p<0.05; and **** p< 0.0001.
