# Supplementary material for: Optogenetics and electron tomography for structure-function analysis of cochlear ribbon synapses
Source: eLife. 2022 Dec 23;11:e79494. doi: 10.7554/eLife.79494 (PMC9908081; doi:10.7554/eLife.79494)
Supplement: Supplementary file 3. — For each ribbon, we determined the cutting plane and classified it as either a cross-section, a longitudinal section, or a section in between both. [file elife-79494-supp3.docx]

**Supplementary file 3**

| **Condition** | ***N_animals_*** | ***n_ribbons_*** | **cross** | **longitudinal** | **between both** |
| --- | --- | --- | --- | --- | --- |
| B6J Light | 2 | 15 | 8 | - | 7 |
| ChR2 Nolight | 4 | 17 | 5 | 5 | 7 |
| ChR2 ShortStim | 1 | 11 | 5 | 5 | 1 |
| ChR2 LongStim | 4 | 26 | 15 | 3 | 8 |

**Cutting planes of the analyzed ribbon synapses for each condition**

For each ribbon, we determined the cutting plane and classified it as either a cross-section, a longitudinal section or a section in between both.
