# Supplementary material for: Optogenetics and electron tomography for structure-function analysis of cochlear ribbon synapses
Source: eLife. 2022 Dec 23;11:e79494. doi: 10.7554/eLife.79494 (PMC9908081; doi:10.7554/eLife.79494)
Supplement: Supplementary file 4. — To judge the size of the tomograms for each condition, we determined the number (and percentage) of tomograms with virtual sections in the most frequent range of 140–170. This range is usually around 50% for all conditions. [file elife-79494-supp4.docx]

**Supplementary file 4**

| **Condition** | **Total # of tomograms** | **# (%) of tomograms with virtual sections 140 -170** | **Number of tomograms with > 140 virtual sections** |
| --- | --- | --- | --- |
| B6J Light | 15 | 10 (**~67 %**) | 2 |
| ChR2 NoLight | 17 | 8 (**47 %**) | 9 |
| ChR2 ShortStim | 11 | 6 (**~55 %**) | 5 |
| ChR2 LongStim | 26 | 13 (**50%**) | 9 |

**The range of virtual sections per condition**

To judge the size of the tomograms for each condition, we determined the number (and percentage) of tomograms with virtual sections in the most frequent range of 140 – 170. This range is usually around 50% for all conditions.
